# Accuracy of transfer learning in predicting peri-implantitis onset from intraoral radiograph: A multi-center longitudinal retrospective study

**DOI:** 10.1007/s00784-026-07181-3

**Published:** 2026-10-19

**Authors:** Muhammad H. A. Saleh, Luigi Nibali, Abdusalam E. Alrmali, Vito Carlo Alberto Caponio, Pasquale Santamaria, Francesco Fanelli, Khrystyna Zhurakivska, Hom-Lay Wang, Giuseppe Troiano

**Affiliations:** 1 Department of Periodontics and Oral Medicine, University of Michigan School of Dentistry, Ann Arbor, MI USA; 2 Centre for Host Microbiome Interactions, Faculty of Dentistry, Oral and Craniofacial Sciences, King’s College London, London, UK; 3 Department of Oral Medicine, Oral Pathology, Oral and Maxillofacial Surgery, University of Tripoli School of Dentistry, Tripoli, Libya; 4 Department of Clinical and Experimental Medicine, University of Foggia, Foggia, 71122 Italy; 5 Department of Medicine and Surgery, LUM University, Casamassima, 70010 Italy

**Keywords:** Artificial intelligence, Peri-implantitis, Dental implants, Machine learning, Retrospective studies

## Abstract

**Background:**

The growing application of artificial intelligence in dentistry has opened new opportunities for predicting peri-implantitis (PI) using convolutional neural networks (CNNs). Transfer learning, where knowledge from a model pre-trained on a large dataset is adapted to a new, domain-specific task, may enhance performance, particularly when medical datasets are limited. This study evaluated the ability of several pre-trained CNN architectures applied to baseline 2D intraoral radiographs to predict peri-implantitis onset.

**Methods:**

A retrospective multi-center study was conducted at the University of Michigan and King’s College London. A total of 195 implants (146 from Michigan and 49 from London) placed in 108 maintained patients were included. For each implant, a baseline periapical radiograph taken up to one year after prosthetic restoration was analyzed. Five pre-trained CNN architectures (ResNet50, DenseNet, VGG16, InceptionV3, EfficientNet) were combined with four transfer learning strategies: feature extraction (hybrid and non-hybrid), partial fine-tuning (50% of layers), and full fine-tuning. Models were trained using patient-wise splits to avoid leakage, and performance was evaluated on an independent test set using accuracy, area under the ROC curve (AUC), sensitivity, and specificity.

**Results:**

All CNN architectures showed variable performance across transfer learning strategies. The non-hybrid feature extraction approach provided the most consistent discrimination, with the best models achieving AUC values between 0.74 and 0.83. InceptionV3 reached the highest AUC (0.83). Fine-tuning strategies yielded modest or low performance, with AUCs generally ranging from 0.42 to 0.69 across architectures.

**Conclusion:**

Non-hybrid feature extraction applied to pre-trained CNNs showed the most promising, though moderate, performance for predicting PI onset from baseline intraoral radiographs. These findings indicate that transfer learning may assist early risk stratification in implant dentistry, but larger prospective studies are needed before clinical adoption.

**Clinical relevance:**

Deep learning models may serve as adjunctive tools to identify implants at higher risk for future PI based on routine baseline radiographs, supporting more individualized maintenance protocols and earlier preventive interventions.

**Supplementary Information:**

The online version contains supplementary material available at https://doi.org/10.1007/s00784-026-07181-3.

## Background

Implant-supported oral rehabilitation is one of the most commonly performed procedures in modern clinical practice [1]. Although dental implants demonstrate high survival and success rates, a considerable proportion develop peri-implantitis (PI) over time, with meta-analyses reporting a weighted mean prevalence of approximately 20%.² [2].

Progressive bone loss is the main trait of PI, resulting in the destruction of the bone surrounding implants [3]. Traditionally, less than 1.5 mm of bone loss at one year after loading is usually regarded as acceptable, with an additional 0.2 mm of loss per year after that [4]; however, the 2018 Consensus Report on the Classification of Periodontal and Peri-Implant Diseases and Conditions reported that progressive bone loss after initial bone remodeling is sufficient for establishing a case definition of PI [5]. Several risk factors have been reported in the literature, such as smoking, surgical procedures, and medical conditions, like osteoporosis and diabetes. Several authors have suggested that radiographic bone quality and quantity may also help predict PI onset [6, 7].

Two-dimensional (2D) radiographs remain the most frequently used imaging modality for assessing peri-implant bone conditions; however, their interpretation is subjective and dependent on operator experience and image quality, which may lead to variability in diagnostic and prognostic decisions [7].

The recent digital transformation era has facilitated the availability of a substantial volume of images derived from clinical practice [8]. These grayscale images can be converted into pixels and subsequently decomposed into data matrix values for further processing to identify patterns correlated with specific clinical events [9].The emergence of deep learning (DL) has profoundly influenced the domain of scientific research, holding the potential to instigate a transformative shift in healthcare delivery and management [10, 11]. In particular, with improvements in the computational capabilities of modern hardware, DL can analyze large amounts of images and patient data to identify patterns and correlations that aid in personalized medicine [12].

In recent years, numerous studies have explored the use of artificial intelligence for implant- and peri-implant–related tasks, which provides the direct background for the present work. AI and DL models have been proposed for automated assessment of peri-implant bone levels and for radiographic prediction of implant failure, using periapical and panoramic images to extract quantitative features linked to adverse outcomes [13]. Other investigations have focused on peri-implantitis detection or diagnosis from 2D radiographs, demonstrating that convolutional neural networks (CNN) can distinguish healthy and diseased implants with promising accuracy, sensitivity, and specificity. In addition, AI has been applied to characterize implant surface or design features and to classify implant systems and prosthetic configurations, parameters that are themselves associated with peri-implant disease risk [14].

Within this evolving technological landscape, transfer learning is a machine learning technique in which knowledge learned from solving one task is applied to a new one by utilizing knowledge previously acquired [15]. It involves using a pre-trained model, trained on a large dataset, and learning to extract general features and patterns from the data. Typically, a smaller dataset can benefit from the transfer of previously acquired knowledge from a larger dataset. A recent literature review has synthesized the various transfer learning approaches into four classes, providing different strategies for medical image classification tasks [16]. Against this background, the present multi-centre retrospective study aimed to test different transfer learning approaches applied to several architectures for predicting PI onset from 2D intraoral radiographs taken after prosthetic insertion in maintained implant patients. The null hypothesis was that transfer learning–based deep learning models would not predict future peri-implantitis onset from baseline intraoral radiographs better than chance.

## Materials and methods

### Study cohort

This retrospective study was performed at the Department of Periodontics and Oral Medicine at the University of Michigan, Ann Arbor, and at the Faculty of Dentistry, Oral and Craniofacial Sciences at King’s College London. The study was approved by the local ethical committees (registration number: IRB00001999 for the Michigan University), and data collected from London practices were part of a service evaluation (reference 14 LO 0629, Nibali et al., 2017) approved by the London City & East NHS Research Ethics Committee. This study was conducted in accordance with the Helsinki Declaration adopted in 1964 and 1975 as revised in 2013 [17]. A retrospective analysis of implants placed from January 2009 to January 2023 in these two centers was performed. The reporting of this observational study was performed according to the Strengthening the Reporting of Observational Studies in Epidemiology (STROBE) guidelines [18].

To be included in the cohort, patients should have fulfilled the following criteria: a partially edentulous area restored with one or more implants and a documented follow-up of at least 2 years after the implant was loaded. Clinical data with the availability of a 2D intraoral radiography performed up to one year post-restoration and at a follow-up of at least 2 years after the prosthetic restoration. To be considered suitable for inclusion, at least 2 mm mesial and distal area to the implant had to be visible; in such a case, the whole intraoral radiography was used as a region of interest (ROI).

The exclusion criteria comprised patients who were undergoing active periodontal therapy at the time of implant placement, those with confounding comorbidities such as uncontrolled diabetes, recent (within the past 2 years) radiation therapy or chemotherapy, psychological issues, or those who had received treatment or maintenance visits from institutions other than the study institutions. All implants that met the aforementioned inclusion criteria were included; therefore, more than one implant per patient was included if all criteria were met.

### Case definitions

#### Peri-implantitis (PI)

Radiographs and patient charts were used to assess the presence/absence of PI according to the definition proposed by the 2017 World Workshop [5, 19]. To be considered as occurring, PI had to onset at any point from baseline to the last follow-up.

**Periodontitis**

Periodontitis was defined according to the case definition proposed by the 2017 World Workshop [5, 19, 20].

#### Radiographic bone loss

Radiographic interproximal bone loss was evaluated as the distance between the most coronal portion of the implant platform and the most coronal radiographic bone-to-implant contact.

### Data extraction

In total, radiographs from 108 patients were included, comprising 195 implants (146 implants in 85 patients from the University of Michigan cohort and 49 implants in 23 patients from the King’s College London cohort). For each implant, a baseline periapical radiograph fulfilling the eligibility criteria was available and used for subsequent analysis. A unified dataset was then created from the two cohorts and later split into training, validation, and test sets. In addition to intraoral radiographs, clinicopathological variables were extracted from clinical records, including sex, age, implant site, date of prosthetic placement, date of last follow-up, and history of periodontitis. Information on systemic conditions was obtained from self-reported questionnaires completed by patients at admission. These questionnaires were updated every 2 years, and the most recent questionnaire at the time of implant placement was used in the present study.

### Images preprocessing and model training

**Fig. 1:**
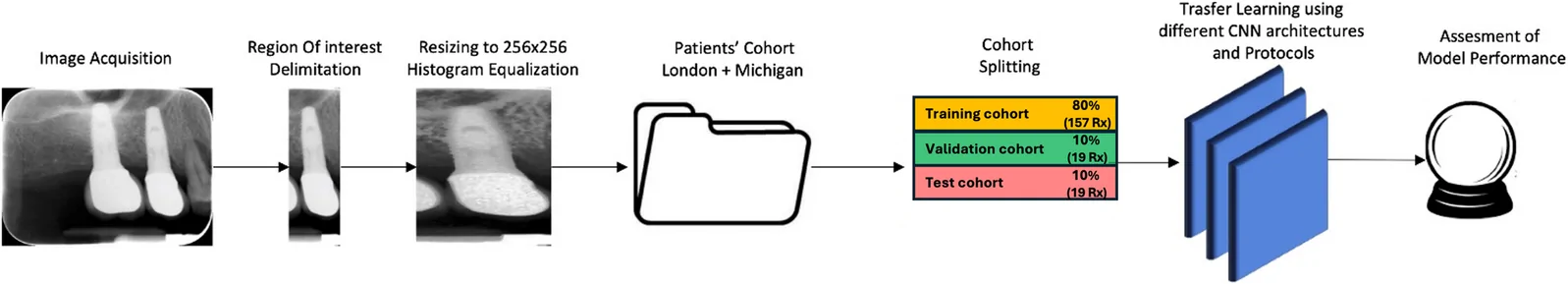
Artificial intelligence workflow starting from image acquisition up to model performance evaluation

Only radiographs at baseline were used for subsequent CNN analysis and underwent an image preprocessing protocol. The ROI was defined to include the area 2 mm mesial and distal to the implant. (Fig. 1). Images were first converted to grayscale and then resized to 256 × 256 pixels. The target size for resizing the images was set to 256 × 256 to maintain a balance between computational efficiency and image quality. These steps were crucial to standardize the images for subsequent analysis and machine learning tasks. In addition, data augmentation techniques, including rotation, zoom, and horizontal flip, were applied to the training set to enhance the model’s capability for generalization.

#### CNN architectures and transfer learning approaches

The selected pre-trained models utilized in this study were: ResNet50, DenseNet, VGG-16, Inception V3, and EfficientNet. By using these CNN architectures, which were previously trained on large-scale image datasets, we aimed to transfer the previously acquired knowledge on the “Image-Net” dataset to the cohort of radiographs included in this study. Four different transfer learning approaches were applied:

i) Feature Extraction Hybrid: The pre-trained CNN models were used as feature extractors. The fully connected layers of the pre-trained models were removed, and a new classifier was added on top of the extracted features. The weights of the pre-trained layers were frozen during training while the new classifier was fine-tuned on the PI dataset.
ii) Feature Extraction Non-Hybrid: This was similar to the Feature Extraction Hybrid mentioned above, but in this case, the entire pre-trained CNN models were used as a fixed feature extractor without adding a new classifier. The extracted features were directly fed into a separate classifier, which was trained from scratch to classify PI images.
iii) Fine Tuning 50%: the third approach involved fine-tuning 50% of the pre-trained CNN model’s layers, while keeping the other 50% frozen. The trainable layers were chosen to be the ones closer to the output layer, while the initial layers were kept frozen to retain the pre-trained knowledge of general image features.
iv) Full Fine-Tuning: In the last approach, the entire pre-trained CNN model was fine-tuned from scratch on the PI dataset. All layers of the pre-trained model were unfrozen, and their weights were updated during training to adapt the model to the PI classification task.

#### Model training, evaluation, and hyperparameter tuning

For each transfer learning approach, the respective CNN model was trained on the training set and evaluated on the validation and test sets. With the aim to model for the hierarchical structure of data, with more implants nested in the same patients, stratified sampling based on patients was employed to split the dataset into training, validation, and test sets, ensuring that all implants from a single patient were exclusively contained within one of these sets. The binary cross-entropy loss function was used for training, and the Adam optimizer was employed for optimization. Hyperparameter tuning was performed to optimize the model’s performance. Different hyperparameters were tested, including learning rate, batch size, and the number of epochs. In this phase, a grouped cross-validation strategy was employed. This technique preserved the integrity of the patient-implant hierarchy within validation folds, offering a refined assessment of the models’ ability to generalize across varied patient conditions. The weighted binary cross-entropy loss function was utilized to account for class imbalance. Furthermore, data augmentation techniques were tailored to reflect the hierarchical data structure, implementing patient-specific transformations. Early stopping was then implemented based on the loss curves during training. If there was no improvement in validation loss after a certain number of epochs (patience), the training process was halted to prevent overfitting and ensure the best model performance on unseen data. Overfitting refers to a situation in which the model fits the training radiographs too closely and therefore performs well on the training set but poorly on new, unseen images. Subsequently, model performance was evaluated on the held-out test set after dataset splitting (80% training, 10% validation, 10% testing). Performance was assessed using classification accuracy, area under the receiver operating characteristic curve (AUC), sensitivity, and specificity; the latter two metrics were calculated using a predicted probability threshold of 0.5. All analyses were performed at the implant level using Python, including TensorFlow and Keras.

## Results

### Participants cohort

**Fig. 2:**
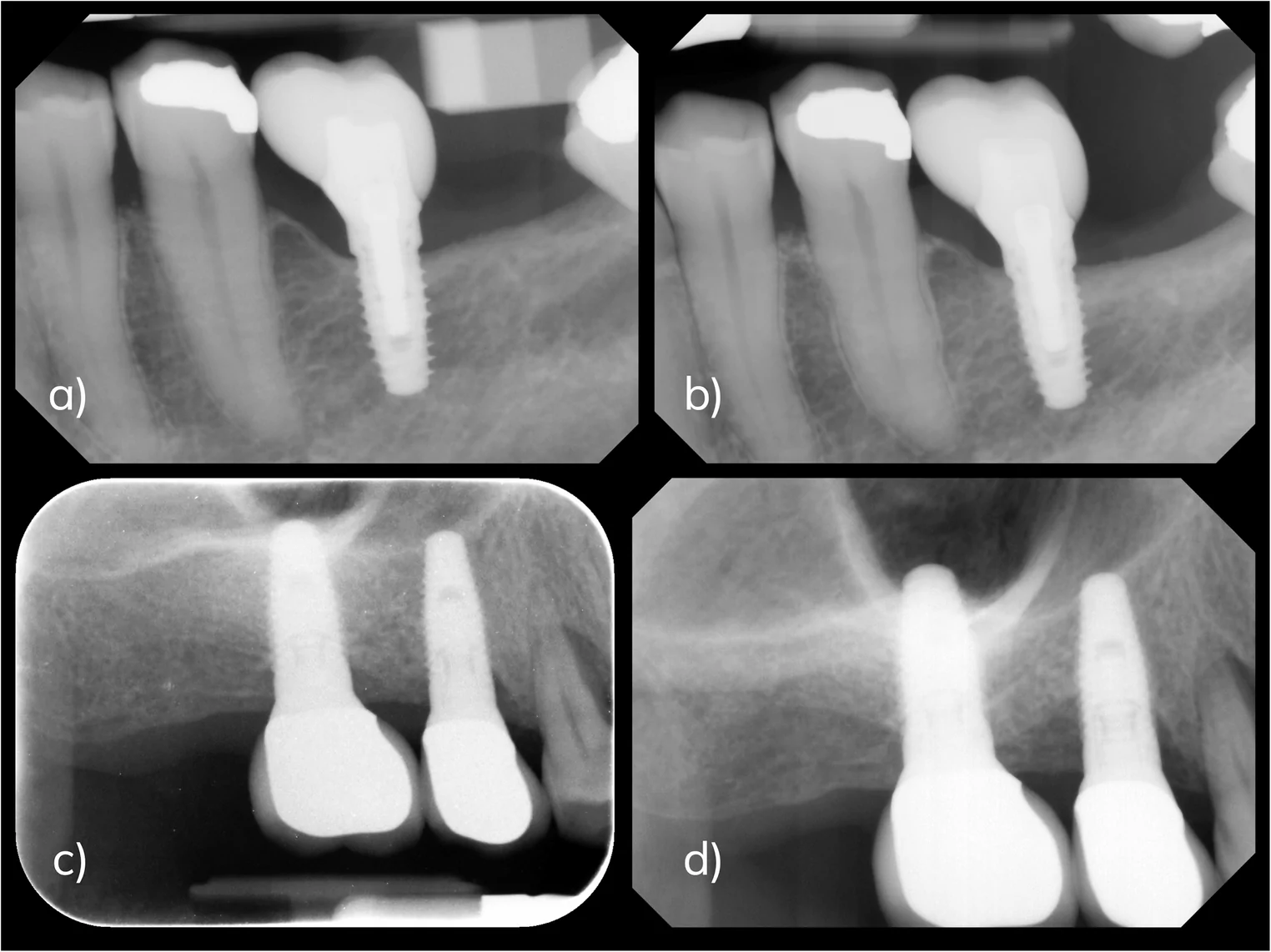
(a-d): (**a**) A baseline and 7 years’ follow-up (**b**) radiography of an implant not developing PI. Another example case of an implant at baseline (**c**) and 6 years’ follow-up (**d**), as shown in the last radiography, the implant developed PI

A total of 195 implants in 108 patients were included in the analysis (146 implants in 85 patients from the Michigan cohort and 49 implants in 23 patients from the London cohort). Of the Michigan implants, 17 in 13 patients were classified as peri-implantitis (PI), and 129 implants in 72 patients were classified as healthy (Fig. 2). In the London cohort, 11 implants in 9 patients were classified as PI, and 38 implants in 20 patients were healthy. The average implant follow-up was 90.59 ± 25.95 months overall. Among the 195 included implants, 119 were placed in the maxilla and 76 in the mandible. The mean age of patients at baseline was 60.73 ± 12.38 years in the Michigan cohort and 56.2 ± 8.7 years in the London cohort. Overall, 78 of 108 patients (72.2%) had a history of periodontitis. Additional details on patient- and implant-level characteristics for both cohorts are provided in Table 1.

**Table 1:** Baseline characteristics of the cohort of patients/implants included in this study. Age, Sex, Smoking, and History of Periodontitis are reported at the patient level, while all the other variables are calculated at the Implant level

Variables	Michigan Cohort	London Cohort
**Sex** Male Female	41/85, 48.24% 44/85, 51.76%	10/23, 43.48% 13/23, 56.52%
**Age (years)**	60.73 ± 12.38	56.2 ± 8.7
**Smoking** Yes No	5/85, 5.88% 80/85, 94.12%	2/23, 8.70% 21/23, 91.30%
**History of Periodontitis** Yes No	61/85, 71.76% 24/85, 28.24%	17/23, 73.91% 6/23, 26.08%
**Marginal bone level at baseline (mm)** Mesial Distal	0.59 ± 0.76 0.58 ± 0.80	0.34 ± 0.55 0.35 ± 0.52
**Marginal bone level at last follow-up (mm)** Mesial Distal	1.07 ± 1.45 1.06 ± 1.38	1.45 ± 1.78 1.47 ± 1.67
**Jaw** Maxilla Mandible	84/146, 57.53% 62/146, 42.47%	35/49, 71.43% 14/49, 28.57%
**Implant Typology** Bone Level Tissue Level	109/146, 74.66% 37/146, 25.34%	47/49, 95.91% 2/49, 4.09%
**Prosthesis** Screwed Cemented	111/146, 76.03% 35/146, 23.97%	44/49, 89.8% 5/49, 10.2%
**Prosthetic Typology** Splinted Single crown	47/146, 32.19% 99/146, 67.81%	23/49, 46.93% 26/49, 53.07%
**Implant Connection** External Internal	26/146, 17.80% 120/146, 82.20%	8/49, 16.32% 41/49, 83.68%

### Models performance

#### Feature extraction approaches

When using the feature extraction hybrid strategy, AUC values varied substantially across architectures, with VGG-16 and DenseNet demonstrating the most stable performance (AUC range 0.67–0.71). The remaining architectures showed limited discriminatory ability under this approach. Full performance metrics for all models are presented in Supplementary Table 1.

With the feature extraction non-hybrid strategy, all architectures achieved moderate and more consistent AUC values, with the best-performing models reaching AUCs in the 0.74–0.83 range. Notably, InceptionV3 achieved the highest AUC (0.83), followed by DenseNet and EfficientNet. Detailed accuracy, sensitivity, and specificity results for all models are provided in Table 2.

**Table 2:** Model performance of the 5 CNN architectures using a feature extraction non-hybrid approach

Model	Feature extraction non-hybrid			
	Accuracy	AUC	Sensitivity	Specificity
VGG-16	0.68	0.59	33.3%	84.6%
Inception-V3	0.74	0.83	66.7%	76.9%
ResNet-50	0.72	0.65	50.0%	76.9%
DenseNet	0.74	0.79	50.0%	84.6%
EfficientNet	0.77	0.74	33.3%	92.4%

### Fine-tuning approaches

Fine-tuning results showed greater variability. Using the 50% fine-tuning strategy, DenseNet achieved the highest AUC (0.68), although overall model discrimination remained modest across architectures. Under this approach, most models displayed unbalanced performance, as detailed in Supplementary Table 2.

For the full fine-tuning strategy, AUC values were generally low across architectures. Among the evaluated models, InceptionV3 and ResNet50 demonstrated similar moderate performance (AUCs 0.63–0.69), while other architectures showed limited predictive ability. Comprehensive performance metrics for Full fine-tuning approaches are summarized in Table 3.

**Table 3:** Model performance of the 5 CNN architectures using a full fine-tuning approach

Model	Full Fine tuning			
	Accuracy	AUC	Sensitivity	Specificity
VGG-16	0.67	0.42	0.0%	94.2%
Inception-V3	0.73	0.69	50.0%	69.7%
ResNet-50	0.71	0.63	16.7%	69.7%
DenseNet	0.71	0.63	16.7%	69.7%
EfficientNet	0.69	0.53	83.3%	30.8%

## Discussion

This multi-center retrospective study showed that transfer learning–based CNN architectures could predict peri-implantitis onset from baseline 2D intraoral radiographs with moderate discriminatory performance. The non-hybrid feature extraction approach provided the most consistent results, with InceptionV3 achieving the highest AUC (0.83), with an accuracy of 0.74, sensitivity of 66.7%, and specificity of 76.9%. These findings suggest that AI-based methods may have potential for predicting peri-implantitis onset from 2D intraoral radiographs. Overall, the non-hybrid feature extraction approach provided the most consistent discriminatory performance across the evaluated CNN architectures. Other approaches, including full fine-tuning, showed promising outcomes in terms of prediction performance. In this study, different CNN architectures and transfer learning approaches were applied to 2D intraoral images obtained within one year after prosthetic restoration. Transfer learning, a concept rooted in human brain behavior, posits that knowledge gained in one context can be applied to another [15]. For instance, the ability to ride a bicycle significantly eases the learning curve of mastering a motorcycle. This principle is also evident in areas such as new language acquisition, pattern recognition, and academic learning [21]. Recently, this concept has been extended to the field of AI, aiming to leverage skills acquired in image recognition for medical image analysis [22]. The assumption is that medical images may share useful visual features with general images, and transfer learning can facilitate knowledge transfer to CNN [23].

Techniques like feature extraction and fine-tuning are among the transfer learning approaches with fewer computationally expensive steps than full fine-tuning. The results of this study can be viewed from both a technical and clinical perspective. Technically, the best performance was achieved with a non-hybrid feature extraction approach. The efficacy of such a method with small datasets, which leverages pre-trained model features without further training, may reduce the risk of overfitting. This highlights its value in medical research, particularly when data scarcity is prevalent. ^8, 9^. Although this approach performed the best, other methods and models also showed acceptable results, suggesting that transfer learning could be applied in daily practice in the future. Our results are in concordance with what is reported for the prediction of implant failure using both radiographic features extracted through CNN models and clinical data [24].

From a clinical standpoint, these findings suggest a practical way to incorporate AI into daily implant maintenance rather than replacing clinical judgment. A model that can flag implants at high risk for future peri-implantitis from a routine baseline intraoral radiograph may help clinicians prioritize recall intervals, tailor oral hygiene reinforcement, and plan earlier supportive or corrective interventions before advanced bone loss occurs. Such tools could be integrated into existing radiographic workflows as decision-support systems, providing a simple risk score or alert that complements conventional risk assessment charts and indices, thereby making AI accessible even to clinicians without formal training in computer science.

From a clinical perspective, these findings could contribute valuable insights into our understanding of PI. Progressive marginal bone loss following prosthetic placement was identified as a diagnostic marker of PI at the 2017 Consensus [5, 19]. However, most of the literature has concentrated on clinical parameters for predicting PI, encompassing anatomical, clinical, and behavioral factors [25]. While some studies have questioned the role of physiological bone remodeling as a phenomenon associated with a higher risk of developing PI later, the topic remains controversial [26–29].

Several recent studies have applied AI to peri-implant imaging, but most have focused on cross-sectional detection or grading rather than longitudinal prediction of future disease. Mugri et al. systematically reviewed AI models for detecting peri-implant bone loss on dento-maxillofacial radiographs and reported accuracies ranging roughly from 0.61 to 0.95, with sensitivities up to about 0.94 and specificities up to 1.00, supporting the diagnostic potential of these tools but also highlighting substantial variability between models and datasets [30]. Lee et al. developed a YOLOv7-based deep learning model to identify implants and classify peri-implantitis severity on periapical radiographs, achieving excellent specificity and F1 scores in a purely detection-oriented setting [14]. Bonfanti-Gris et al. summarized AI applications for implant classification and peri-implant pathology identification in 2D radiographs and concluded that, although accuracies for pathology detection were generally above 0.79, many studies suffered from risk of bias and limited external validity [13]. In contrast to these primarily diagnostic or cross-sectional approaches, the present study uses transfer learning on baseline intraoral radiographs in a multi-center longitudinal cohort to predict subsequent peri-implantitis onset, positioning AI not only as a tool to detect established disease but also as an adjunct for early risk stratification and preventive decision-making in maintained implant patients.

Interestingly, the application of DL in the current study leveraged the use of 2D radiographical images to predict the later onset of PI. This suggests that something not readily recognizable in clinical observation may already be present at the time of prosthetic insertion, indicating the potential existence of pathology in some cases. These latent features offer valuable insights into the early stages of disease development, often preceding visible clinical symptoms. In the realm of disease prediction, it is known that pathological changes may commence beneath the surface, in the bone or soft tissues, before becoming visible. Machine learning models can capture these subclinical changes by extrapolating from baseline data, effectively “anticipating” PI development based on early, subvisual cues. The promising performance assessed in this study suggests that the model may indirectly capture variations in bone anatomy on X-rays, which could be influenced by host-related factors such as smoking, PPI usage, diabetes, and auto-immune diseases. Drawing from related research by some authors of this manuscript, it was observed that mesial and distal sites of the same implant were clustered together in 95.45% of patients, reinforcing the model’s reliability [31]. These observations indicate that patient- or site-specific bone features, as reflected in radiographic images, might provide insights into underlying molecular and genetic characteristics [32]. However, it is essential to note that explicit identification of these host-related factors was beyond the scope of our current study.

AI models can automatically generate features not immediately apparent to human observers. These engineered features encapsulate intricate relationships within the data, including subtle changes over time, forming a basis for predicting future disease onset. Another potential application of AI is to enhance existing risk assessment tools like the Implant Disease Risk Assessment [33]. Integration of information gathered from 2D intraoral radiographs could further improve the accuracy and comprehensiveness of such risk assessment tools.

While the results are promising, this study has some limitations. Firstly, the relatively limited number of patients and implants included from the two participating centers may limit the generalizability of the findings. Other limitations include the diverse skill levels and expertise among different categories of providers contributing to the collected data, as well as the use of various implant systems, each separately associated with the prevalence of PI [34, 35]. The study also encompasses different prosthetic designs, some of which are considered risk factors for PI [27, 36]. Moreover, this study primarily relies on radiographic assessments, neglecting soft tissue variables such as keratinized mucosa width, mucosal thickness, or peri-implant soft tissue height [37]. Additionally, due to the 2D nature of the radiographs, only the interproximal aspects could be assessed, omitting the possibility of evaluating buccal thread exposure.

This study, while offering valuable insights into predicting peri-implantitis using DL, faces limitations including its retrospective nature, limited sample size, and reliance on data from two university cohorts, potentially affecting generalizability. The exclusive use of radiographic analysis omits crucial soft tissue assessments and varies by implant system, which could influence outcomes. Additionally, the focus on radiographic features neglects clinical indicators of inflammation, crucial for a comprehensive peri-implantitis diagnosis. These constraints underscore the necessity for broader, more inclusive research to validate and refine predictive models for peri-implantitis. Lastly, the study is retrospective in design, inherently lacking the benefits of randomization and prospective observation. The present findings need to be confirmed in future studies on the topic to gain a deeper understanding of the potential of AI methods for the diagnosis and early prediction of PI.

## Conclusion

Our study demonstrates the promising potential of AI, particularly CNN and transfer learning techniques, in predicting peri-implantitis onset from 2D intraoral radiographs. This holds significant implications for improving peri-implantitis prediction models in implant dentistry. Further research in larger cohorts is necessary to validate and refine these AI-based prediction models for sound clinical applications.

## Supplementary Information

Below is the link to the electronic supplementary material.

Supplementary Material 1

Supplementary Material 2

## Data Availability

The data used to support the findings of this study are available from the corresponding author upon request.
