# Supplementary material for: Accuracy of transfer learning in predicting peri-implantitis onset from intraoral radiograph: A multi-center longitudinal retrospective study
Source: Clin Oral Investig. 2026 Oct 19;30(11):537. doi: 10.1007/s00784-026-07181-3 (PMC13677619; doi:10.1007/s00784-026-07181-3)
Supplement: Supplementary file 1 [file 784_2026_7181_MOESM1_ESM.docx]

| Model | Feature Extraction Hybrid | | | |
| --- | --- | --- | --- | --- |
|  | Accuracy | AUC | Sensitivity | Specificity |
| VGG-16 | 0.79 | 0.67 | 50.0% | 92.3% |
| Inception-V3 | 0.52 | 0.53 | 16.6% | 69.2% |
| ResNet-50 | 0.63 | 0.46 | 0.0% | 92.3% |
| DenseNet | 0.74 | 0.71 | 33.3% | 92.3% |
| EfficientNet | 0.53 | 0.52 | 100.0% | 0.0% |

Supplementary Table 1. Model performance of the 5 CNN architectures using a feature extraction hybrid approach.
