# Supplementary material for: Accuracy of transfer learning in predicting peri-implantitis onset from intraoral radiograph: A multi-center longitudinal retrospective study
Source: Clin Oral Investig. 2026 Oct 19;30(11):537. doi: 10.1007/s00784-026-07181-3 (PMC13677619; doi:10.1007/s00784-026-07181-3)
Supplement: Supplementary file 2 [file 784_2026_7181_MOESM2_ESM.docx]

| Model | Fine Tuning 50% | | | |
| --- | --- | --- | --- | --- |
|  | Accuracy | AUC | Sensitivity | Specificity |
| VGG-16 | 0.68 | 0.50 | 0.0% | 100.0% |
| Inception-V3 | 0.41 | 0.50 | 100.0% | 0.0% |
| ResNet-50 | 0.41 | 0.50 | 100.0% | 0.0% |
| DenseNet | 0.73 | 0.68 | 16.7% | 100.0% |
| EfficientNet | 0.53 | 0.51 | 16.7% | 76.9% |

Supplementary Table 2. Model performance of the 5 CNN architectures using a fine-tuning 50% approach.
